# Malignant germ cell tumours of the testis express interferon-*γ*, but are resistant to endogenous interferon-*γ*

**DOI:** 10.1038/sj.bjc.6601209

**Published:** 2003-08-26

**Authors:** S Schweyer, A Soruri, J Peters, A Wagner, H J Radzun, A Fayyazi

**Affiliations:** 1 Division of Pathology, Department of Pathology, University of Göttingen, Robert-Koch Str. 40, D-37075 Göttingen, Germany; 2 Department of Immunology, University of Göttingen, Robert-Koch Str. 40, D-37075 Göttingen, Germany; 3 Department of Cardiovascular Physiology, University of Göttingen, Robert-Koch Str. 40, D-37075 Göttingen, Germany

**Keywords:** TGCT, cytokine, apoptosis

## Abstract

Cytokines possess discrepant effects on tumour cells varying from anti- to proapoptotic activities. We recently reported that testicular germ cell tumours (TGCT) express a functional form of the proinflammatory cytokine interferon-gamma (IFN*γ*). The present study asked whether TGCT-derived IFN*γ* influences survival or death of neoplastic germ cells. Analysis of TGCT cell lines demonstrated that they expressed and secreted IFN*γ*, but were resistant to the endogenous IFN*γ* since neutralisation of IFN*γ* by a specific blocking antibody had no influence on the proliferation and/or the degree of apoptosis of tumour cells. To study mechanisms providing tumour resistance to endogenous IFN*γ*, we analysed primary TGCT and two human TGCT cell lines (NTERA and NCCIT) for the expression of IFN*γ* receptor and for the level of phosphorylation of the signal transducer and activator of transcription (STAT)-1. *In situ* hybridisation, immunocytochemistry, Western blot analysis and flow cytometry indicated that primary TGCT as well as NCCIT and NTERA cell lines expressed the heterodimeric cell surface IFN*γ* receptor which consists of both 90-kDa *α*- and the 85-kDa *β*-chains. However, the downstream transcription factor STAT-1 was not phosphorylated constitutively, indicating that STAT-1 is not activated by the endogenous IFN*γ*. Upon application of recombinant human IFN*γ* in excess, however, STAT-1 was phosphorylated and the interferon regulatory factor-1 (IRF-1) was induced, suggesting that both IFN*γ*R and STAT-1 are functionally intact in TGCT. Altogether our results suggest that despite secreting biologically active IFN*γ*, the concentration of the endogenous IFN*γ* is too low to stimulate the IFN*γ*R/STAT signalling pathway in TGCT in an autocrine and/or paracrine manner.

Interferon-*γ* (IFN*γ*) is a pleiotropic cytokine mainly secreted by activated T lymphocytes and natural killer (NK) cells (Mosman and Sad, 1996). The cellular response to IFN*γ* is mediated by a heterodimeric cell surface receptor (IFN*γ*R), which consists of two subunits, the 90-kDa *α* chain (IFN*γ*R*α*) and the 85-kDa *β* chain (IFN*γ*R*β*) (Valente *et al*, 1992; Pestka *et al*, 1997). Ligation of IFN*γ* to the *α* chain clusters the neighbouring *β* chain of the IFN*γ*R followed by Janus kinase-mediated phosphorylation of the signal transducer and activator of transcription (STAT)-1, the homodimer of which migrates to the nucleus and stimulates gene transcription such as the interferon regulatory factor-1 (IRF-1) (Haque and Williams, 1998; Wagner *et al*, 2002).

After the first report of its ability to protect cells from viral infection, IFN*γ* has been demonstrated to play a crucial role in host defence, inflammation and autoimmunity. For instance, IFN*γ* has been shown to augment antigen presentation by upregulation of major histocompatibility complex class I and II molecules, to induce proinflammatory cytokines in effector cells and to orchestrate leukocyte–endothelium interaction by upregulation of adhesion molecules (Boehm *et al*, 1997). Regarding induction of apoptosis, however, IFN*γ* seems to play a paradoxical role in different normal and neoplastic cell types. Whereas in normal macrophages and neoplastic myeloid and NK cells, IFN*γ* prevents apoptosis (Lotem and Sachs, 1996; Mizuno *et al*, 1999; Xaus *et al*, 1999), it enhances apoptosis of tumour cells in malignancies such as pancreatic carcinoma, colon carcinoma and ovarian carcinoma (Adachi *et al*, 1999; Burke *et al*, 1999; Detjen *et al*, 2001).

Testicular germ cell tumours (TGCT) are the most common solid malignancy in young males from 20 to 40 years old and represent a heterogeneous group of different histological entities composed of seminomatous and nonseminomatous tumours (Ulbright, 1993) that are almost all infiltrated by various numbers of T lymphocytes (Torres *et al*, 1997). In an attempt to investigate mechanisms navigating T lymphocytes into the TGCT, we observed that neoplastic germ cells express IFN*γ* (Schweyer *et al*, 2002). Based on this evidence and considering the dual role of IFN*γ* in apoptosis of neoplastic cells, we asked whether TGCT-derived IFN*γ* possesses any effect on survival or death of TGCT.

## MATERIALS AND METHODS

### Tissue samples

Tumour specimens were obtained from 12 patients who underwent orchiectomy for testicular tumour. Patients had not received any chemotherapeutic or immunomodulatory treatment before operation. The mean age at the time of operation was 36.8 years, ranging from 28 to 55 years. Tumour tissues were classified according to the classification system of the World Health Organization (six cases of pure seminoma, six cases of combined tumour containing nonseminomatous and seminomatous components) (Mostofi, 1980). Probes from normal testes were obtained from three patients who underwent bilateral orchiectomy because of prostatic cancer. Two blocks of each testis were immediately frozen in liquid nitrogen and stored at −80°C until analysed by reverse transcription–polymerase chain reaction (RT–PCR). Samples of each tissue specimen were also fixed in neutral formalin and processed for histology, immunohistochemistry and *in situ* hybridisation (ISH).

### Testicular germ cell tumour cell lines

The human TGCT cell lines used in this study were NTERA (American Type Culture Collection, ATCC, Manassas, VA, USA; CRL-1973) and NCCIT (ATCC, CRL-2073). The cell lines were grown as monolayers and maintained in HEPES-buffered RPMI 1640 (Biochrom, Berlin, Germany) supplemented with 10% fetal calf serum (FCS) (CC Pro, Neustadt, Germany), 100 IU ml^−1^ penicillin (Sigma, Deisenhofen, Germany), 100 *μ*g ml^−1^ streptomycin (Sigma) and 2 mM L-glutamine (Life Technologies, Karlsruhe, Germany). Cultures were incubated at 37°C in a humid atmosphere with 5% carbon dioxide in air.

### Human monocytes

Blood monocytes were obtained from healthy volunteers as described previously (Schweyer *et al*, 2002). A total of 2.5 × 10^5^ monocytes were cultured in basis medium containing RPMI 1640, 2 mM L-glutamine, 1% penicillin and streptomycin, and 15% human serum for 1 day. The cells were then stimulated for 12 h by recombinant human IFN*γ* (rhIFN*γ*; R&D Systems) at a final concentration of 200 U ml^−1^ before being analysed for the expression of IFN*γ*R*α* and IFN*γ*R*β* and for the phosphorylation of STAT-1 (see below).

### Human umbilical vein endothelial cells

Human umbilical vein endothelial cells (HUVEC) were isolated and cultured as described previously (Schweyer *et al*, 2002). The cells were then stimulated for 9 h by rhIFN*γ* (1000 U ml^−1^) and rhTNF*α* (100 U ml^−1^) before being studied for the expression of IRF-1 as described elsewhere (Wagner *et al*, 2002).

### Proliferation and apoptosis of TGCT cell lines following IFN*γ* neutralisation

NCCIT and NTERA cells (1 × 10^6^) were cultured in 96-well round-bottom microtitre plates (Nunc, Wiesbaden, Germany) at 37°C in 5% CO_2_ atmosphere in the presence of a specific neutralising polyclonal antibody against human IFN*γ* (R&D Systems, Heidelberg, Germany) in excess (100, 500 or 1000 *μ*g ml^−1^) or in the presence of control isotype-matched IgG (100, 500 or 1000 *μ*g ml^−1^, Dako, Hamburg, Germany) for 24 or 48 h.

To assess the proliferation activity of NCCIT and NTERA cells, plates were pulsed with 1 *μ*Ci per well of [^3^H]thymidine. After 24 or 48 h, the cells were collected by an automated Inotech cell harvester (Dunn, Ansbach, Germany) and the radioactivity was measured with a *β*-counter (Hewlett-Packard, Meriden, CT, USA). Results were expressed as counts per minute (c.p.m.)±s.e.m. Each experiment was performed in triplicate and was repeated three times.

To determine the apoptotic rate of NCCIT and NTERA cells, they were harvested at 24 or 48 h after application of neutralising antibody or control IgG. Then the cells underwent May–Giemsa–Grunwald (MGG) staining, or immunocytochemistry for the active form of caspase-3, or *in situ* end labelling (ISEL) for DNA fragmentation. For control, NCCIT cells were incubated with 50 *μ*M cisplatin (Holzkirchen, Germany) for 24 h and proved to be apoptotic as described previously (Burger *et al*, 1999). Each experiment was performed in triplicate and was repeated three times.

### May–Giemsa–Grunwald staining

Centrifuged cells (2 × 10^3^) were dried for 24 h, fixed in 100% acetone for 10 min, stained with MGG and embedded in ‘SuperMount Medium’. Apoptotic cells were identified by cellular shrinkage and nuclear condensation and fragmentation.

### Immune staining

#### Antibodies

The monoclonal antibody against placental-like alkaline phosphatase (PLAP, clone 8A9) and the monoclonal antibody against cytokeratine (clone MNF116) were obtained from Dako (Hamburg, Germany); the monoclonal antibody against IFN*γ*R*α* (clone GIR-94), the polyclonal antibody against IFN*γ*R*β* (C-20) and the polyclonal antibody against pStat-1 (Tyr701) were from Santa Cruz Biotechnology (Heidelberg, Germany); and the polyclonal antibody against IFN*γ* (AF-285-NA) and the polyclonal antibody recognising the active form of caspase-3 were from R&D systems (Wiesbaden, Germany). The antibodies were applied at a working dilution of 1 : 25 (IFN*γ*) or 1 : 50 (PLAP, cytokeratine, IFN*γ*R*α*, IFN*γ*R*β*, pSTAT-1 and active caspase-3).

#### Immunohistochemistry

Immunohistochemical reactions for IFN*γ*R*α* and IFN*γ*R*β* were performed on frozen sections. After incubation with the primary antibody, the sections were incubated with a horseradish peroxidase (HRP)-conjugated biotin–streptavidin amplified system (Dako) and the signals were visualised with 3,3′-diaminobenzidine (DAB; Dako) as described previously (Fayyazi *et al*, 2000). Immunohistochemical reactions for PLAP and cytokeratin were performed on paraffin-embedded serial sections. After incubation with the primary antibody, the sections were incubated with an AP-conjugated or an HRP-conjugated biotin–streptavidin amplified system (Dako) and the signals were visualised with fast red or AEC (Dako) as described elsewhere (Schweyer *et al*, 2000). All samples were counterstained with Meyer's haematoxylin, mounted in Super Mount Medium and analysed by light microscopy. Controls were stained as above omitting primary or secondary antibodies.

#### Immunofluorescence and immunocytochemistry

Fresh tumour tissue was mechanically dissociated in a suitable volume of RPMI 1640 supplemented with 100 IU ml^−1^ penicillin, 100 mg l^−1^ streptomycin and 2 mM L-glutamine. Cells were spun down at 1500 r.p. m. for 5 min and the pellet was resuspended in 500 *μ*l ice-cold phosphate-buffered saline (PBS). Then, 5 × 10^5^ cells were cytocentrifugated on slides coated with 2% 3-aminopropyltriethoxy-silane, dried for 24 h and fixed with 100% acetone for 10 min at room temperature. For immunocytochemical staining, cells were incubated for 30 min with a primary antibody against IFN*γ*, IFN*γ*R*α*, IFN*γ*R*β*, active form of caspase-3 or p-STAT-1. To visualise bound primary anti-IFN*γ*-antibody, cells were incubated with an FITC-labelled rabbit anti-goat IgG (working dilution 1 : 500) for 1 h (Dako), mounted with ‘Fluorescent Mounting Medium’® and examined using fluorescence microscopy. To visualise bound primary anti-IFN*γ*R*α*, anti-IFN*γ*R*β*, anti-active caspase-3 and p-STAT-1 antibodies, cells were incubated with AP-conjugated or HRP-conjugated biotin–streptavidin amplified system (Dako) as described previously (Schweyer *et al*, 2000). Fast red or AEC (Dako) was applied as chromogen. After signal development, cells were counterstained with Meyer's haematoxylin, mounted in Super Mount Medium and analysed by light microscopy. IFN*γ*-stimulated human monocytes served as positive control.

### *In situ* end labelling (ISEL)

Fixed centrifuged cells (2 × 10^3^) were incubated with TBS (50 mM Tris-HCl; 150 mM NaCl; pH 7.5) containing 10% FCS and 0.3% H_2_O_2_ for 15 min. The cells were then incubated for 60 min at 37°C with 50 *μ*l of the labelling mix (250 U ml^−1^ terminal transferase, 20 *μ*l ml^−1^ Digoxigen-DNA labelling mix at 10 × concentration and 1 mmol l^−1^ CoCl_2_ in reaction buffer for terminal transferase (Roche, Mannheim, Germany)). After rinsing in TBS, the cells were blocked with 10% FCS and incubated for further 60 min with a rabbit HRP-conjugated F(ab)_2_ fragment against digoxigenin (working dilution: 1 : 500, Dako). 3,3′-Diaminobenzidine was next applied as chromogen. Cells with fragmented DNA revealed nuclear brown signals. DNA-fragmented cells with intact plasma membrane were considered to be apoptotic. Negative controls were stained as above but without terminal transferase.

### Identification and quantification of apoptosis and statistical analysis

Apoptotic cells were identified by nuclear condensation and fragmentation in MGG staining, by positive cytoplasmic signals for the active form of caspase-3 in immunocytochemistry or by DNA fragmentation in ISEL. All experiments were performed in triplicate and were repeated three times with similar results.

Percentage of apoptotic cells was calculated as the ratio of apoptotic cells to 500 cells counted. Results are expressed as the mean ±s.e.m. The differences were analysed with a *t*-test and were considered significant at *P*<0.05.

### RNA extraction and RT–PCR

Total RNAs were extracted from TGCT cell lines as well as from IFN*γ*-stimulated human monocytes using the Qiagen RNA isolation kit (Qiagen, Hilden, Germany), digested with DNAse I and transcribed to cDNA using oligo-d(T) primers and SuperScript II reverse transcriptase (RT) (Life Technologies). In brief, 1–5 *μ*g of total cellular RNA was incubated for 50 min at 37°C with 50 U of RT and 20 U placental RNase Inhibitor in a 20-*μ*l volume containing 2.5 *μ*mol l^−1^ oligo-d(T) primers, 5 mmol l^−1^ MgCl_2_, 50 mmol l^−1^ KCl, 10 mmol l^−1^ Tris-HCl and 1 mmol l^−1^ of each of the deoxynucleoside-triphosphate, heated to 70°C and subsequently cooled to 5°C.

The quantity and quality of the extracted RNA were determined by spectrophotometry and agarose gel electrophoresis. Polymerase chain reaction amplification of *β*-actin cDNA was performed on each sample as a control for efficient cDNA synthesis. Negative controls, which were performed without cDNA adjunction in the reagent mixture, were included for every PCR analysis. Specific primers were custom synthesised (MWG, Ebersberg, Germany) and specific fragments were amplified using primer pairs as follows: IFN*γ*, 5′-GATGACCAGAGCATCCAAAAGAG-3′, 5′-GCAT-CTGACTCCTTTTTCGCTTC-3′; IFN*γ*R*α*, 5′-CCGAAGACAATCCAGGAAAA-3′, 5′-GGAGGTGGGGGCTTTTATTA-3′; IFN*γ*R*β*, 5′-GGAGGAATCCAACAGGTCAA-3′, 5′-CAGACGTCATCCTTTG-GTGA-3′. The PCR reaction mixture consisted of 1.5 mmol l^−1^ MgCl_2_, 50 mmol l^−1^ KCl, 10 mmol l^−1^ Tris-HCl, 0.2 *μ*mol l^−1^ each of both the primers and 2.5 U of *Taq* DNA polymerase (Pharmacia, Freiburg, Germany) in a 0.1 ml volume containing 20 *μ*l of the above-mentioned RT reaction mixture. Reaction conditions were: 2 min at 96°C followed by 35 cycles of 30 s at 95°C, 45 s at specific annealing temperature (IFN*γ* 56°C, IFN*γ*R*α* 58°C, IFN*γ*R*β* 58°C) and 45 s at 72°C, and a final extension at 72°C for 10 min. The amplified products were electrophoresed in ethidium bromide-stained 1.5% agarose gel.

### Subcloning of IFN*γ*, IFN*γ*R*α* and IFN*γ*R*β* cDNA, preparation of cRNA probes and nonradioactive ISH

For preparation of riboprobes, fragments of the human IFN*γ*, IFN*γ*R*α* and IFN*γ*R*β* cDNA were subcloned into pBluescript II KS+ phagemid (Stratagene, CA, USA). The subcloned fragment served as template for *in vitro* transcription of digoxigenin-11-dUTP labelled antisense and sense probes, which were generated by virtue of T3- and T7-polymerase according to the manufacturer's instructions (Roche).

*In situ* hybridisation for IFN*γ* mRNA was performed according to the method described previously (Schweyer *et al*, 2002). After signal detection, specimens were subjected to double-staining by indirect immunofluorescence for TGCT markers PLAP and cytokeratin, as described above (Schweyer *et al*, 2002). To detect and visualise IFN*γ*R transcripts, the Catalyzed Signal Amplification System was used as recommended by the manufacturer (Dako). DAB (Dako) was applied as chromogen. After signal detection, specimens were mounted in Super Mount Medium. For each tissue specimen, sense riboprobes were applied as controls and proved to be negative.

### Flow cytometry

NCCIT-, NTERA- and IFN*γ*-stimulated human monocytes were spun down at 1500 r.p. m. for 5 min and the pellet was resuspended and washed in 500 *μ*l of ice-cold TBS. The cell suspension was then centrifuged again at 1500 r.p.m. and resuspended in 10 ml RPMI 1640 cell culture medium. Cells were then transferred to 96-well round-bottom microtitre plates (Nunc, Wiesbaden, Germany), which had been precoated by blocking buffer (10% heat-inactivated rabbit serum and 0.1% NaN_3_ in PBS). Thereafter, they were incubated for 30 min with a monoclonal antibody against human IFN*γ*R*α*, a polyclonal antibody against IFN*γ*R*β* or irrelevant mouse/rabbit IgG (Dako). After washing with PBS, the cells were incubated for further 30 min with an FITC-conjugated goat anti-mouse or a PE-conjugated goat anti-rabbit antibody (Dako). Cells were then washed again, fixed with 2% paraformaldehyde and subjected to flow cytometric analysis using FACStar^plus^ (Becton Dickinson, San Jose, CA, USA).

### Western blot

For immunoblot analysis, TGCT cell lines were harvested in 500 *μ*l of lysis buffer (0.4% sodium deoxycholate, 1% NP-40, 50 mM EGTA, pH 7.4, 10 mM Tris pH 7.4, 1 mM phenylsulphonyl fluoride (PMSF), 10 *μ*g ml^−1^ leupeptin, 10 *μ*g ml^−1^ aprotinin). The resulting lysates were resolved on 10% sodium dodecyl sulphate–polyacrylamide gel electrophoresis (SDS–PAGE) gels and transferred onto nitrocellulose membranes (Schuett, Göttingen, Germany) or polyvinylidene fluoride transfer membrane (Pall Corporation, Roßdorf, Germany). The membranes were blocked with Tris-buffered saline with Tween 20 (10 m Tris-HCl, pH 7.4, 150 mM NaCl, 0.1% Tween 20) containing 5% milk and then incubated with the antibodies against IFN*γ*R*α*, IFN*γ*R*β*, pSTAT-1 or IRF-1 (Santa Cruz). The working dilution of antibodies used for Western blots was 1 : 2000. Proteins were detected using enhanced chemiluminescence (ECL) reagents (Amersham Pharmacia Biotech, Freiburg, Germany). Loading and transfer of equal amounts of protein in each line was verified by reprobing the membrane with a monoclonal anti-*β*-actin antibody (Sigma). Lysates of IFN-stimulated HeLa cells (Santa Cruz) served as positive control.

### ELISA

Extracellular IFN*γ* level in culture supernatants of TGCT cell lines was measured via commercial ELISA (R&D) as recommended by the manufacturer. The lower limit for assay was IFN*γ* <3 pg.

## RESULTS

### Testicular germ cell tumours express IFN*γ*

**Figure 1:**
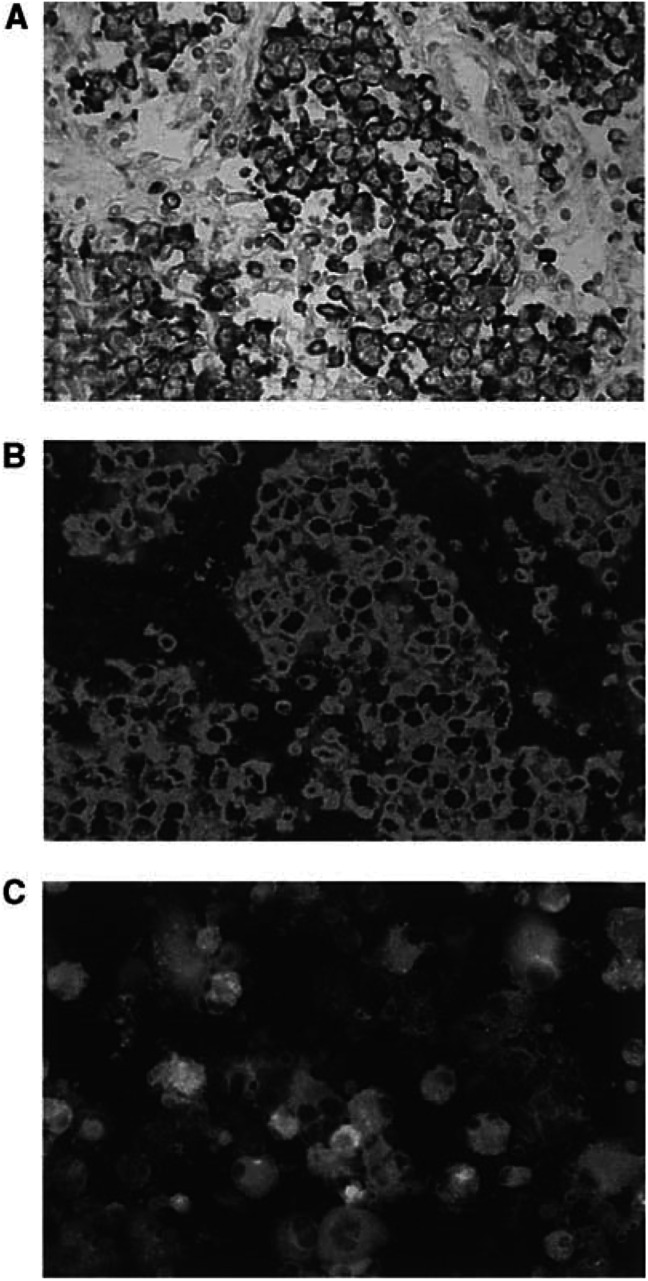
Expression of IFN*γ* in primary TGCT (**A**–**C**). Nonradioactive ISH reveals IFN*γ* transcripts in a representative TGCT with seminomatous differentiation (**A**, black signals). Combination of ISH with immunofluorescence shows that IFN*γ*+ cells are neoplastic germ cells expressing the seminoma marker PLAP (**B**, green signals). Immunocytochemistry illustrates the expression of IFN*γ* protein in isolated neoplastic germ cells of the same tumour (**C**, green signals).

To determine the expression and cellular localisation of IFN*γ* in TGCT, *in situ* analyses were performed. Whereas no IFN*γ* expression was noted within the normal testes (data not shown), nonradioactive ISH revealed that numerous tumour-infiltrating mononuclear cells and almost all neoplastic germ cells independent of their histological type expressed IFN*γ* mRNA (Figure 1

). Consequently, we asked whether IFN*γ* mRNA is translated into IFN*γ* protein. To answer this question, tumour cells were isolated from TGCT and subjected to immunofluorescence. Results demonstrated that primary TGCT not only produce IFN*γ* mRNA but also IFN*γ* protein (Figure 1).

For an autocrine effect, IFN*γ* must be secreted by tumour cells. To prove this, we analysed two well-established human TGCT cell lines NCCIT and NTERA, for the expression and secretion of IFN*γ*. Reverse transcription–polymerase chain reaction RT–PCR showed that both cell lines expressed IFN*γ* mRNA (data not shown). Applying ELISA, significant amounts of secreted IFN*γ* (343–112 pg ml^−1^) were found in culture supernatants of NCCIT and NTERA, as described previously (Schweyer *et al*, 2002) (data not shown). Based on this background, we next asked whether TGCT-derived IFN*γ* influences the proliferation and/or apoptosis of the testicular tumour cells in an autocrine manner.

### Testicular germ cell tumour-derived IFN*γ* does not affect proliferation or apoptosis of TGCT cell lines

**Figure 2:**
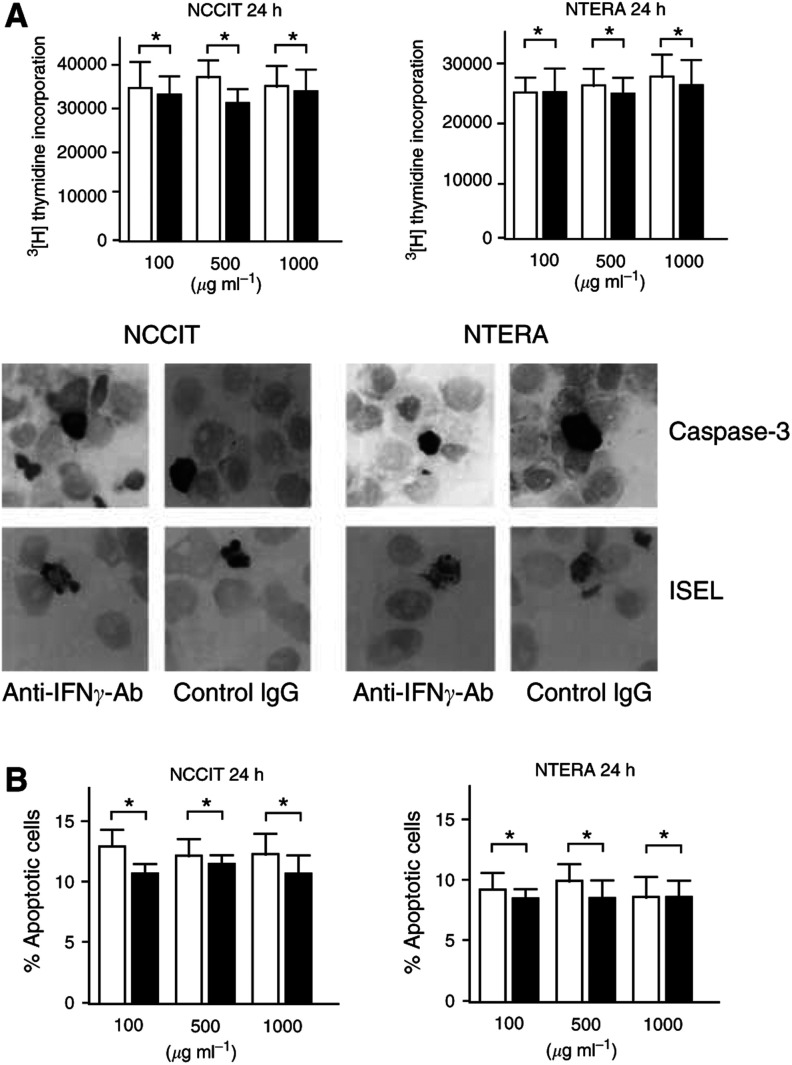
Proliferation and apoptosis of two TGCT cell lines (**A** and **B**). NCCIT and NTERA cells were incubated with different concentrations of a neutralising antibody (Ab) against human IFN*γ* (100, 500 or 1000 *μ*g ml^−1^) or with control isotype-matched IgG (100, 500 or 1000 *μ*g ml^−1^). After 24 h, the proliferation activity and the apoptosis rate of the cells were assessed by [^3^H]thymidine assay and morphological methods, respectively. The [^3^H]thymidine assay does not reveal any significant difference in the proliferation activity of NCCIT or NTERA cells following IFN*γ* neutralisation (white columns) when compared with controls (black columns). Results are expressed as counts per minute (c.p.m.)±s.e.m. (**A**). To prove the effect of IFN*γ* neutralisation on apoptosis of NCCIT and NTERA cells, they were stained with MGG for the detection of nuclear condensation and fragmentation, with immunocytochemistry for the active form of caspase-3 and with ISEL for DNA fragmentation. Representative photomicrographs of active caspase-3 (black cytoplasmic signals) and of DNA fragmentation (black nuclear signals) illustrate no significant difference in apoptosis rate of cells incubated with the anti-IFN*γ* Ab (1000 *μ*g ml^−1^) or incubated with control IgG (1000 *μ*g ml^−1^) (**B**). The diagrams show quantification of tumour cell apoptosis with MGG staining following application of anti-IFN*γ* Ab (white columns) or control IgG (black columns) (**B**). Each experiment was performed in triplicate and was repeated three times. The values given (^*^P>0.05, Student's *t*-test) are for the statistical significance of the difference between the two groups.

To study the effect of the secretory IFN*γ* on multiplication and/or death of TGCT cells, endogenous IFN*γ* was blocked by adding different concentrations of a neutralising antibody to the cultured NCCIT and NTERA cells for 24 or 48 h. Results from the [^3^H]thymidine assay demonstrated that IFN*γ* has no effect on proliferation of the TGCT cell lines when compared to controls. In accordance, immunocytochemistry for the active form of caspase-3, ISEL for DNA fragmentation and MGG staining for the detection of nuclear condensation and fragmentation showed that although the apoptosis rate of tumour cells was increased following IFN*γ* neutralisation when compared to controls, the differences among the groups remain, however, not significant (*P*>0.05). Figure 2

demonstrates results from proliferation and apoptosis assays on TGCT cell lines 24 h after IFN*γ* neutralisation. Similar results were also seen 48 h after beginning IFN*γ* neutralisation (data not shown).

### Testicular germ cell tumours express both IFN*γ*R subunits *α* and *β*

Since it is known that IFN*γ* mediates its effects by binding to a specific high-affinity receptor (Boehm *et al*, 1997), we asked whether the unresponsiveness of TGCT to the endogenous IFN*γ* is due to the lack or dysfunction of IFN*γ*R on neoplastic germ cells.

**Figure 3:**
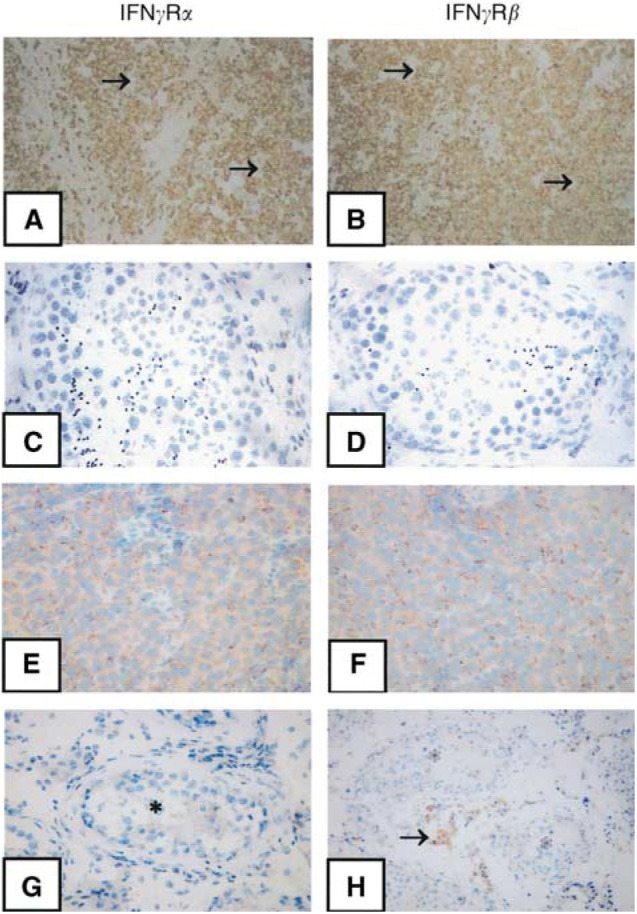
Expression of IFN*γ*R in primary TGCT and normal testis (**A**–**H**). Nonradioactive ISH demonstrates IFN*γ*R*α* mRNA (**A** and **C**, brown signals) and IFN*γ*R*β* mRNA (**B** and **D**, brown signals) in a representative TGCT with seminomatous differentiation (**A** and **B**) and in a representative normal testis (**C** and **D**). Immunostaining shows the expression of IFN*γ*R*α* (**E** and **G**, brown signals) and IFN*γ*R*β* proteins (**F** and **H**, brown signals) in the same tumour (**E** and **F**) and in the same normal testis (**G** and **H**). Note that neoplastic but not normal germ cells in tubuli seminiferi (asterisks) express both IFN*γ*R*α* and IFN*γ*R*β*. Also note that, in the normal testis, Leydig cells (arrow) express IFN*γ*R*β*, but not IFN*γ*R*α*.

**Figure 4:**
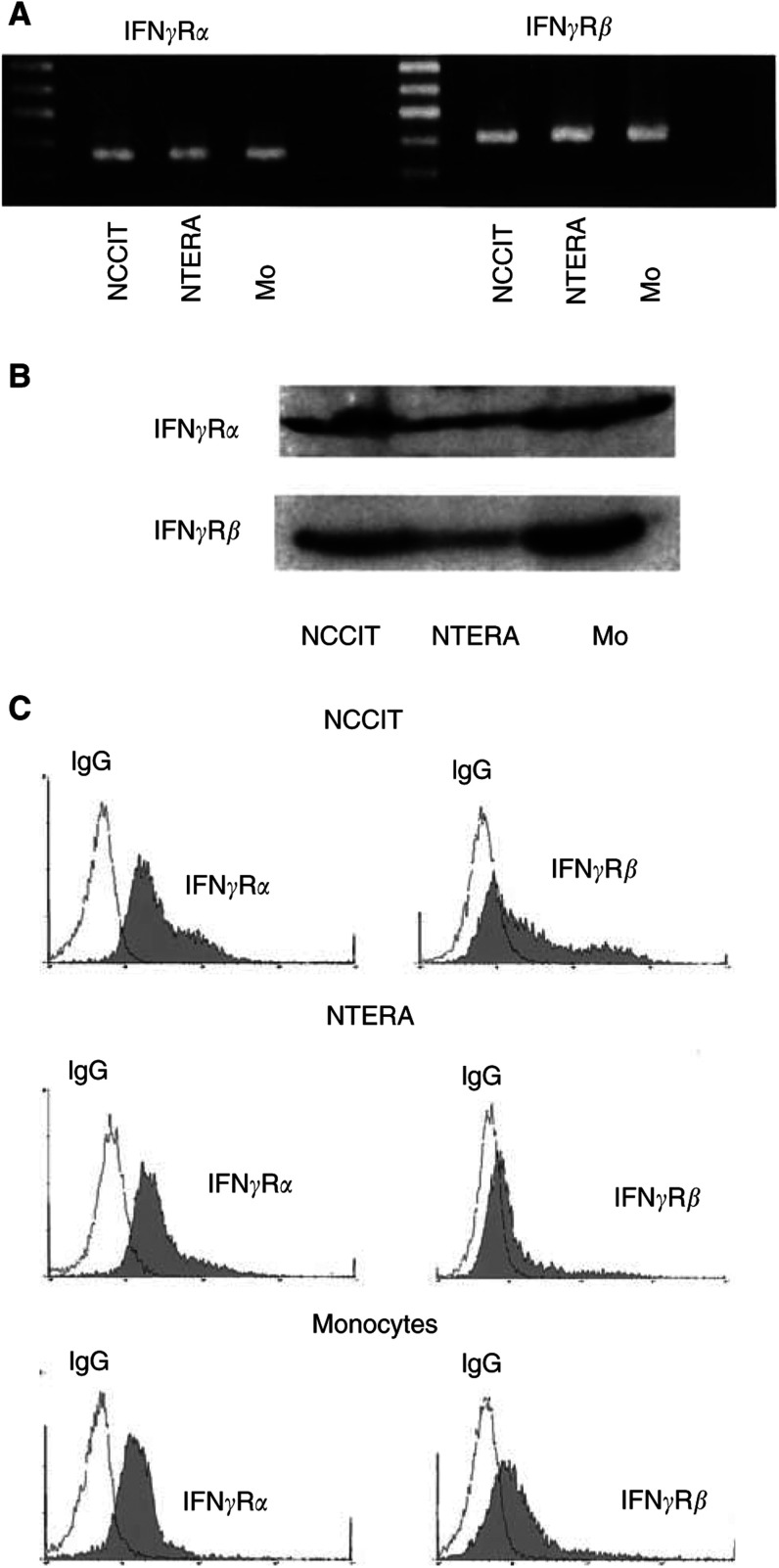
Expression of IFN*γ*R proteins in TGCT cell lines (**A**–**D**). Production of IFN*γ*R*α* and IFN*γ*R*β* was examined by RT–PCR (**A**), Western blot (**B**) and FACS analysis (**C**). IFN*γ*R*α* and IFN*γ*R*β* mRNA (**A**) and proteins (**B** and **C**) are expressed in both TGCT cell lines NCCIT and NTERA. For control, human monocytes (Mo) were treated with IFN*γ*, as described in Materials and Methods, and proved to express IFN*γ*R*α* and IFN*γ*R*β* mRNA (**A**) and proteins (**B** and **C**). In part C, cells were stained by indirect immunofluorescence with anti-IFN*γ*R*α* or anti-IFN*γ*R*β* antibody (filled histograms) or with isotype control IgG (open histograms).

Nonradioactive ISH revealed that in addition to numerous tumour-infiltrating mononuclear cells, almost all tumour cells independent of their histological type expressed IFN*γ*R*α* and IFN*γ*R*β* mRNAs (Figure 3

). Consequently, the expression of IFN*γ*R*α* and IFN*γ*R*β* proteins in primary tumours was examined by immunohistochemistry and immunocytochemistry. Results illustrated both IFN*γ*R subunits on neoplastic germ cells in primary TGCT as well as on the surface membrane of tumour-infiltrating mononuclear cells (Figure 3). Within the normal testes, however, no expression of IFN*γ*R*α* or IFN*γ*R*β* was noted in germ cells (Figure 3). *In situ* hybridisation and immunohistochemistry, however, indicated that IFN*γ*R*β*, but not IFN*γ*R*α*, was expressed in Leydig cells (Figure 3). In addition to primary tumours, both human TGCT cell lines were also examined for the IFN*γ*R expression. RT–PCR, Western blot and FACS analyses demonstrated that NCCIT and NTERA cell lines express IFN*γ*R*α* and IFN*γ*R*β* (Figure 4

).

### Testicular germ cell tumours lack STAT-1 activation

**Figure 5:**
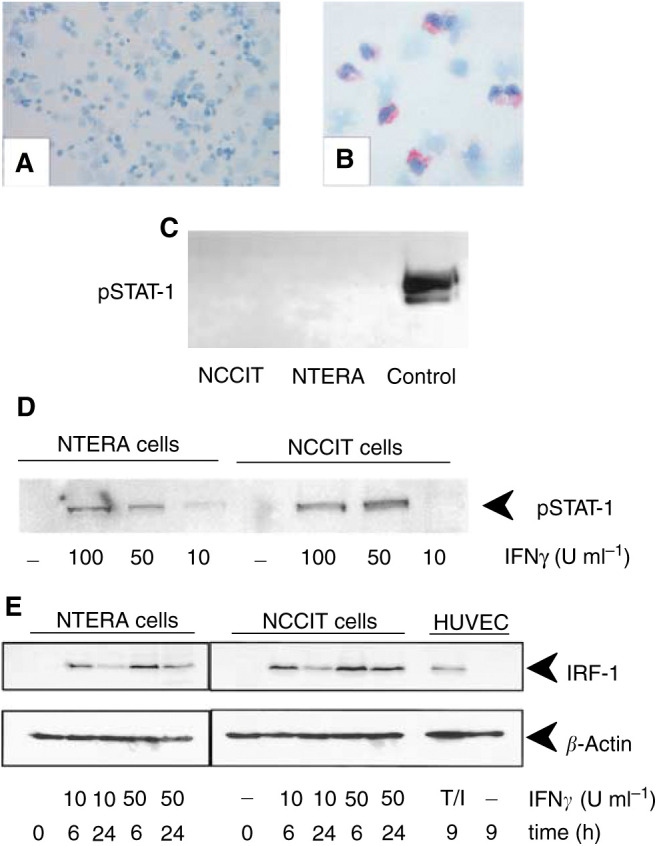
Expression and function of STAT-1 protein in TGCT (**A**–**E**). Production of phosphorylated STAT-1 (pSTAT-1) protein was studied in primary TGCT as well as in TGCT cell lines. Immunocytochemistry (**A** and **B**) and Western blot (**C**) demonstrate the lack of pSTAT-1 in tumour cells from a representative TGCT with seminomatous differentiation (**A**) and in NCCIT and NTERA cell lines (**C**). At 24 h after application of rhIFN*γ* (10, 50 or 100 U ml^−1^), however, STAT-1 was phosphorylated (**D**) and IRF-1 was induced in both TGCT cell lines (**E**). IFN*γ*-stimulated monocytes (**B**, red signals), commercially purchased lysates of stimulated HeLa cells (**C**) or HUVECs costimulated with rhIFN*γ* and rhTNF*α* (**E**) served as positive controls for immunocytochemistry and Western blots, respectively.

After demonstration of the IFN*γ*R expression on TGCT, the functional activity of the IFN*γ*R was analysed. For this, we examined whether the transcription factor STAT-1 is phosphorylated in IFN*γ*-expressing neoplastic germ cells because it is known that a sufficient stimulation of IFN*γ*R results in a STAT-1 activation through phosphorylation. Immunocytochemistry on tumour cells isolated from primary TGCT and Western blot analysis of NCCIT and NTERA cell lines revealed that STAT-1 is not constitutively phosphorylated in neoplastic germ cells (Figure 5

). To examine whether the IFN*γ*R/STAT signaling pathway is intact in TGCT cells, we stimulated NCCIT and NTERA cell lines with different doses of rhIFN*γ*. Western blot analysis demonstrated that upon stimulation STAT-1 was phosphorylated and IRF-1 was induced in both cell lines (Figure 5), suggesting that both IFN*γ*R and STAT-1 are biologically intact.

## DISCUSSION

In the present study, we investigated the effect of IFN*γ* on proliferation and apoptosis of TGCT. Analyses showed IFN*γ* in almost all neoplastic germ cells of primary TGCT. The data presented in this report extended our previous findings (Schweyer *et al*, 2002) because they demonstrated that primary TGCT not only expressed IFN*γ* mRNA but also IFN*γ* protein. These findings were surprising, because IFN*γ* is normally expressed and secreted by inflammatory leucocytes (Elgert *et al*, 1998) but not by tumour cells. Moreover, IFN*γ* is primarily known as a cytokine with several antitumour properties. For instance, it has been shown that IFN*γ* possesses direct cytotoxic effects on ovarian carcinoma cell lines (Kim *et al*, 2002), augments apoptosis-inducing capacity of TNF*α* in cervical carcinoma cells (Suk *et al*, 2001), reduces the proliferation activity of colon carcinoma cells and melanoma cells (Raitano and Korc, 1993; Krasagakis *et al*, 1995), and is able to upregulate MHC molecules on renal cell carcinomas, thus leading to a better recognition of neoplastic cells by cytotoxic T cells (Totpal and Aggarwal, 1991; Hillman *et al*, 1997). In addition to these antitumour activities, however, IFN*γ* seems to have also some powerful protumour effects. For instance, it is well known that IFN*γ* is a potent inhibitor of apoptosis in some haematological malignancies (Lotem and Sachs, 1996; Mizuno *et al*, 1999).

Based on this background, we hypothesised that endogenous IFN*γ* affects the proliferation and/or apoptosis of neoplastic germ cells. To prove this hypothesis, we first analysed two human TGCT cell lines for the expression and secretion of IFN*γ*. Results demonstrated that both NCCIT and NTERA cell lines produce and release significant amounts of IFN*γ*. Next, we neutralised the IFN*γ* in culture supernatants of the TGCT cell lines by applying a specific antibody to study the role of secretory IFN*γ* on the neoplastic germ cells. Using independent proliferation and apoptosis assays, we did not however note any evidence showing that the endogenous IFN*γ* influences the multiplication and/or the death rate of TGCT cells. These findings, however, must be strengthened by *in vitro* experiments with neoplastic cells isolated from primary tumours.

Taking into account that the TGCT-derived IFN*γ* is biologically active, because it induces the c-x-c chemokine IP-10 in cultured endothelial cells, as shown in our previous report (Schweyer *et al*, 2002), and considering the fact that IFN*γ* mediates its effects through a high-affinity receptor consisting of a ligand-binding polypeptide chain *α* and a signal-transducing chain *β* (Pestka *et al*, 1997), and many tumours (e.g. hepatocellular carcinoma, prostatic carcinoma, basal cell carcinoma) do not express both receptor chains, thus providing tumour resistance to IFN*γ* (Kooy *et al*, 1998; Nagao *et al*, 2000; Royuela *et al*, 2000), we proved whether the TGCT unresponsiveness to endogenous IFN*γ* is due to the absence of IFN*γ*R. Applying nonradioactive ISH and immunohistochemistry, however, IFN*γ*R mRNA and protein for both *α* and *β* chains were detected in primary TGCT. Immunoblots and flow cytometry revealed that not only primary tumours but also TGCT cell lines express both receptor chains on their cell surface. Based on the fact that TGCT express IFN*γ*R and stimulation of the IFN*γ*R results in activation of downstream transcription factor STAT-1, we studied the level of STAT-1 phosphorylation in neoplastic germ cells. Analysis of primary tumours and cell lines indicated that the transcription factor STAT-1 is not constitutively phosphorylated/activated in TGCT. For this phenomenon, we considered three possibilities. Firstly, the concentration of the endogenous IFN*γ* may be too low to stimulate the IFN*γ*R; secondly, IFN*γ*R is functionally inactive, as demonstrated in renal cell carcinomas (Dovhey and Ghosh, 2000); and, finally, STAT-1 lacks TGCT, as shown in pul-monary carcinoma and malignant melanoma cells (Kaplan *et al*, 1998; Lee *et al*, 1999). Taking these possibilities into account, we stimulated the cell lines with rhIFN*γ* and examined whether STAT-1 was phosphorylated and, if yes, whether pSTAT-1 acts as a functional transcription factor eliciting the expression of interferon-regulated proteins such as IRF-1. Results demonstrated that upon application of rhIFN*γ* in excess (on an average five times the concentration of endogenous IFN*γ* measured in supernatants of the TGCT cell lines), STAT-1 was phosphorylated and IRF-1 was induced. Thus, we suggest that IFN*γ*R and STAT-1 are biologically intact in TGCT, but the level of the endogenous IFN*γ* is not able to activate the IFN*γ*R/STAT signalling pathway in an autocrine and/or paracrine manner. Despite the lack of direct influence on neoplastic germ cells, an outstanding question may be whether endogenous IFN*γ* alters the stromal microenvironment in TGCT, including enhancement of angiogenesis, modification of extracellular matrix composition, recruitment of inflammatory cells and dysbalance of protease activity and thereby the tumour development and progression.
